# Healthcare-Associated Infections Controller: Concept Development

**DOI:** 10.1590/0034-7167-2023-0443

**Published:** 2025-03-10

**Authors:** Karolayne Cabral Matias, Cyntia Leenara Bezerra da Silva, Hanna Priscilla da Silva Medeiros, Maria Isabel da Conceição Dias Fernandes, Dândara Nayara Azevêdo Dantas, Lays Pinheiro de Medeiros, Allyne Fortes Vitor

**Affiliations:** I Universidade Federal do Rio Grande do Norte. Natal, Rio Grande do Norte, Brazil; II Hospital Universitário Ana Bezerra. Santa Cruz, Rio Grande do Norte, Brazil

**Keywords:** Concept Formation, Infection Control, Cross Infection, Nursing, Delivery of Health Care, Formación de Concepto, Control de Infecciones, Infección Hospitalaria, Enfermería, Atención a la Salud

## Abstract

**Objective:**

To develop the concept of a Healthcare-Associated Infections Controller.

**Method:**

A qualitative methodological study, based on the Hybrid Model of Concept Development, conducted in three phases: Theoretical Phase (through a scoping review with a sample of 28 studies), Field Phase (online meeting with 30 professionals in the field), and Final Analytical Phase (data analysis and concept definition).

**Results:**

The antecedents, attributes, and consequences identified in the theoretical phase were similar to those described by the professionals. In the field phase, the majority of professionals defined “Infection Controller” as the most appropriate term for this professional. In the final analytical phase, the elements identified in the previous phases were similar; however, they had particularities.

**Final considerations:**

The concept of a Healthcare-Associated Infections Controller was developed, providing nurses with a better understanding of their role in care delivery and enhancing their awareness of their actions.

## RESUMEN

**Objetivo:**

Desarrollar el concepto de Controlador de Infecciones Relacionadas con la Atención de Salud.

**Método:**

Estudio metodológico cualitativo, basado en el Modelo Híbrido de Desarrollo de Conceptos, realizado en tres fases: Fase teórica (mediante una scoping review, con una muestra de 28 estudios), Fase de campo (reunión en línea con 30 profesionales del área) y Fase analítica final (análisis de los datos y definición del concepto).

**Resultados:**

Los antecedentes, atributos y consecuencias identificados en la fase teórica fueron similares a los descritos por los profesionales. En la fase de campo, la mayoría de los profesionales definieron al “Controlador de infecciones” como el término más adecuado para este profesional. En la fase analítica final, los elementos identificados en las fases anteriores son similares, sin embargo, presentan particularidades.

**Consideraciones finales:**

Se desarrolló el concepto de Controlador de Infecciones Relacionadas con la Atención de Salud, proporcionando a los enfermeros una mejor comprensión de su rol en la atención prestada y una mayor concienciación sobre sus acciones.

## INTRODUCTION

To effectively meet patient needs, it is essential to provide quality healthcare, focusing on well-being and safe care. Poor quality of care and compromised patient safety increase the risk of various complications, such as Healthcare-Associated Infections (HAIs). Globally, HAI affect approximately 1.5 million people annually, making them a major health issue worldwide, with one of the main concerns being the reduction of their incidence^(1,2)^.

When considering the quality of services related to the control and prevention of HAI, it becomes evident that healthcare professionals have a significant impact on the safety of the care provided, highlighting the need for knowledge of their actions^(3,4)^.

In the context of HAI, Ministry of Health Ordinance No. 2.616/1998 recommends the preferential inclusion of a nurse among the members implementing these guidelines, as this profession works continuously in direct patient care, performing invasive and potentially contaminated procedures. Consequently, nurses bear greater responsibility for the prevention and control of infections^(5,6)^.

Although this topic is widely discussed in the literature regarding its origins and the general actions for the prevention and control of HAI, there remains a lack of understanding and a significant conceptual gap regarding the role of the HAI controller in Brazil. This may be contributing to the difficulty in recognizing infection control nurses within their field of practice. Given the above and the identified problem, the following guiding questions arise: What are the conceptual definitions of a HAI Controller? And, finally, what are the antecedents, attributes, and consequences of the concept of a HAI Controller?

## OBJECTIVE

To develop the concept of a HAI Controller.

## METHODS

### Ethical aspects

The study received approval from the Research Ethics Committee of the Federal University of Rio Grande do Norte (UFRN). The participation of the infection controllers was formalized through the acceptance and online signing of the Informed Consent Form (ICF), with clear and secure information provided regarding their rights. Privacy and confidentiality of the collected data were respected.

### Type of study

This is a methodological study with a qualitative approach, grounded in the theoretical-methodological framework of the Hybrid Model of Concept Development, proposed by Schwartz-Barcott and Kim^(7)^. This model involves an interaction between theoretical analysis and empirical observation in field research, allowing the researcher to formulate and refine terminologies in their initial phase of development, as well as to detect conceptual inconsistencies and errors. The final product is the definition of the concept. The model includes three phases, which the authors suggest should overlap during development: theoretical phase, field phase, and final analytical phase^(7)^.

### Methodological procedures

As a strategy in the theoretical phase for data collection and analysis from the literature, a Scoping Review was conducted using the JBI methodological framework^(8)^ and the PRISMA model to guide the entire reporting of results, ensuring greater scientific rigor in the review. The review was operationalized through a research protocol. A preliminary literature search was conducted to verify whether previous review studies on the same topic existed, with none being identified.

The PCC strategy was applied – Population (healthcare professionals), Concept (Controller), and Context (HAI on a global scale) – and by correlating the key topics with the proposed objectives, the following guiding research questions were outlined: What is the origin, description, and context of the concept of Controller? What words or expressions are used in the literature to describe the concept of an HAI Controller? What are the antecedents, attributes, and consequences of the concept of an HAI Controller?

The descriptors “Hospital Infection; Cross Infection,” “Infection Control; Infection Control,” “Nursing; Nursing,” and “Healthcare; Delivery of Health Care” were identified after consultation with the Health Sciences Descriptors (DeCS) and Medical Subject Headings (MeSH). Data were collected from the following electronic databases: Latin American and Caribbean Health Sciences Literature Database (LILACS), SCOPUS (Elsevier), PubMed, and Cumulative Index to Nursing, accessed via the Federated Academic Community (café in Portuguese), through the Coordination for the Improvement of Higher Education Personnel (CAPES) journal portal. Notably, advanced search strategies were employed for all combinations.

For study selection, the following inclusion criteria were established: full-text access, listed in the selected databases, addressing the concept of an HAI Controller, and written in English, Portuguese, or Spanish. Opinion articles, letters to the editor, and editorials were excluded from the study.

Through database searches, 1,331 studies were identified. Of these, 291 were excluded due to duplication. As a result, 982 articles were screened by title and abstract, of which 924 were removed for not meeting the eligibility criteria. Thus, 58 studies were read in full. No additional articles were added through reverse search. In the end, 28 studies comprised the final review sample.

After being tabulated in an Excel® database, the results were exported to the Statistical Package for the Social Sciences 22.0 (SPSS) and analyzed using simple descriptive statistics. All results were presented in tables and charts.

### Study Setting

An analysis of the existing resumes on the Lattes Platform, from the National Council for Scientific and Technological Development (CNPq in Portuguese) portal, was conducted, and the snowball sampling technique was used to select participants. Recruitment was done through invitations sent via a messaging app (WhatsApp), with the goal of having participants join a meeting to evaluate the information gathered during the theoretical phase. Participants were instructed to click on a link that redirected them to a Google® Forms page containing ICF in electronic format.

### Data source

Healthcare professionals specializing in the control of HAIs, who serve as executive members of the HAI control service across its three levels, were invited to participate, as it was believed that the success of the consensus group technique depends on the qualification of the participants^(9)^. Healthcare professionals who were not part of the HAI Control Service or those who served on the HAI Control Commission only as consultants were excluded from the study. A total of 30 professionals participated: 26 nurses, 3 nursing technicians, and 1 administrative assistant from the Hospital Infection Control Committee (CCIH in Portuguese). During the recruitment process, 80 individuals declined to participate in the study.

### Data collection and organization

Data collection took place in February 2023, remotely, through the Google Meets platform, allowing HAI controllers from different parts of the country to participate in the meeting. A link was sent to participants, redirecting them to a Google Forms page containing the authorization form for voice and image recording.

The data collection instrument consisted of a structured presentation covering the following points: 1. Analysis of the expressions found in the literature to define this professional; 2. Analysis of the developed concept; 3. Analysis of the antecedents of the concept; 4. Analysis of the attributes of the concept; 5. Analysis of the consequences of the concept.

The meetings were guided by the researcher, who explained each step so that the controllers could reach a consensus on each theoretical item identified in the previous phase, allowing for close observation by the researcher. Data analysis was descriptive, with transcription and review by the researcher. Items that achieved 100% consensus among the controllers were considered validated. The results were presented in tables and charts. Qualitative data analysis was conducted using the content analysis technique. Participants were identified by codes ranging from C1 to C30 to ensure that no information could reveal their identities.

### Final Analytical Phase

Although the process was presented linearly, the analytical phase permeated the entire research development. The previous phases were regularly evaluated in detail to ensure the methodological reliability and rigor that the model requires. In this way, the integration of the phases made it possible to construct a conceptual framework for the Controller phenomenon, which was then presented in textual form.

## RESULTS

### Theoretical Phase

In this phase, the origin, definitions, antecedents, attributes, and consequences of the concept of the HAI Controller were investigated through a scoping review, resulting in a final sample of 28 studies.

The analyzed studies revealed a growing trend in publications over the past 10 years, with a prevalence between 2018 and 2022 (39.3%). It was also found that the majority of the studies were conducted in Brazil (96.4%) and 89.3% were published in Portuguese.

Regarding the origin of the concept of the HAI Controller, it was possible to identify not only the nature of the concept’s use but also the key elements that enabled its evolution over time. As evidenced, the pursuit of quality in healthcare services associated with HAIs is the primary driving force behind the emergence of the concept (25%).

The concept definitions investigated during this phase served as the foundation for the development of the concept, alongside the multiprofessional perspective of HAI controllers, which was further developed in the field phase. Chart 1 presents a summary of the HAI Controller concept published in the literature.

**Chart 1:** Summary of the definitions of the HAI Controller concept found in the studies from the scoping review, Natal, Rio Grande do Norte, Brazil, 2023

Definitions of HAI Controller	Number of studies	Identification of references
Responsible for supervising standards and routines, training staff and professionals, rationalizing the use of antimicrobials, providing epidemiological information, and minimizing the incidence of HAIs.	11	1, 4, 5,7, 9, 11, 14, 15, 16, 19, 24
Implementer of infection prevention and systematic control measures, reducing risks and harm, ensuring safe management.	8	2, 3, 20, 21, 22, 23
Responsible for identifying and diagnosing different types of hospital-acquired infections, along with maintaining best practices in prevention, control, and information on infections.	5	6, 8, 13, 17, 27
Developer of measures that standardize the organization of actions and health services, in alignment with the principles and guidelines for infection prevention and control, enabling the reduction of their iatrogenic potential.	3	10, 18, 28
Manager of the work process, planning and executing actions to control HAI and assisting in improvement efforts in the area.	1	12

It was observed that the studies selected for the review used seven distinct expressions to describe the concept of Controller, namely: “Supervisor” (21.4%); “Responsible for Prevention” (17.9%); “Risk and Damage Manager” and “Infection Potential Minimizer” (14.3% each); “Infection Controller,” “Strategist for Implementing Best Practices,” and “Identifier of Susceptibilities and Vulnerabilities” (10.7% each). It is noteworthy that all the identified expressions showed similarity in their actions.

With the development of the scoping review analysis, it became necessary to determine the contextual aspects (practical and population-based) that contributed to the interpretation and characterization of the concept. It was observed that the concept is most frequently used in the hospital context (53.6%).

The essential elements of the concept were derived from four antecedents, three attributes, and three consequences. Table 1 shows the categorization of these essential elements.

**Table 1:** Essential elements of the Controller concept identified in the reviewed literature. Natal, Rio Grande do Norte, Brazil, 2023

Essential elements of the HAI Controller concept	n	%
Antecedents		
Few professionals specialized in HAI control	14	50
Low adherence of professionals to safety protocols	8	28.6
Increase in infection rates	4	14.3
Risk to patient safety	2	7.1
Attributes		
Provision of health education for HAI prevention and control	14	50
Implementation of HAI prevention and control guidelines	8	28.6
Identification of infection risks, diagnosis, and case reporting	6	21.4
Consequences		
Improvement in the quality of healthcare	13	46.4
Reduction and control of HAIs	9	32.1
Increased professional knowledge of their role	6	21.4

The most frequent antecedent of the Controller concept is the shortage of professionals specialized in HAI control (50%). The main attribute found in the literature was the “provision of health education for the prevention and control of HAIs” (50%). The improvement in the quality of healthcare was identified as the main consequence (46.4%).

With a clearer theoretical framework, it was possible to proceed to the field phase more precisely. Additionally, the following definition of the HAI Controller was developed to guide professionals in refining the concept: “A duly trained healthcare professional responsible for implementing measures to prevent and control HAI, promoting strategies to minimize infection rates in accordance with guidelines, to ensure the quality of healthcare and patient safety.”

### Field Phase

The field research involved the participation of 26 nurses (86.7%), three nursing technicians (10%), and one administrative assistant from the CCIH (3.3%). Of the professionals who participated in the study, 28 (93.3%) were female, with an average age of 35.9 years. Regarding academic background, 21 (70%) had some type of specialization, and the average time since graduation was 10.3 years. In terms of professional experience, seven (23.3%) reported working in Natal, Rio Grande do Norte, with an average of 5.5 years of experience. Table 2 details the characteristics of the study participants.

**Table 2:** Characteristics of the professionals who participated in the study. Natal, Rio Grande do Norte, Brazil, 2023

Variables	n		%
Profession			
Nurse	26		86.7
Nursing technician	3		10
Administrative assistant	1		3.3
Total	30		100%
Gender			
Female	28		93.3
Male	2		6.7
Total	30		100%
Academic background			
Specialization	11		36.7
Undergraduate degree	9		30
Master's degree	7		23.3
Technical course	3		10
Total	30		100%
Variables	Média	Desvio padrão	Mediana
Age*	35.9	9.3	33.5
Time since graduation*	10.3	8.0	8.5
Length of professional experience*	5.5	4.7	4.5

*Legend: In years.*

In practical terms, all professionals reported that they currently work or have worked in hospitals (100%). In the population context, the Hospital Infection Control Service stands out, with 12 professionals (40%), followed by the Hospital Infection Control Commission, with 10 professionals (33.3%). None of the professionals reported working or having worked in virtual environments. Regarding the participants’ familiarity with the study subject, 29 professionals (96.7%) reported having heard the term “Controller” before.

The concept elements found in the literature to define the HAI Controller were presented to the professionals during this data collection phase. Regarding the selected expressions, 20 participants (66.7%) believed that “Infection Controller” is also an appropriate term to define this type of professional; 11 (36.7%) selected the options “Supervisor” and “Strategist for implementing best practices,” 10 (33.4%) chose “Risk and damage manager,” and 4 (13.4%) chose “Responsible for prevention.” Thus, by consensus, the participants chose the expression “Infection Controller” as the most appropriate for the concept.

The antecedents, attributes, and consequences observed during this phase are described in Chart 2. The elements identified in this phase, similar to those identified in the theoretical phase, are presented without emphasis. New elements or changes that emerged from the participants’ understanding and expressed meanings are highlighted in bold.

**Chart 2:** Essential elements of the Controller concept identified in the field research. Natal, Rio Grande do Norte, Brazil, 2023

**ANTECEDENTS**	Few professionals specialized in HAI control
	Low adherence of professionals to safety protocols
	Increase in infection rates
	Risk to patient safety
**ATTRIBUTES**	Provision of health education for the prevention and control of HAIs
	Implementation and management of the HAI Control Program
	Identification of infection risks, diagnosis, and case reporting
**CONSEQUENCES**	Improvement in the quality of healthcare
	Reduction and control of HAIs
	Increased professional knowledge of their role
	Cost reduction
	Adaptation of hospital infrastructure
	Increase in cleanliness of the environment

In the final evaluation of the concept, it was observed that the elements identified in the previous phases are similar; however, they present particularities that were discussed throughout the study. Through the field phase, it was possible to corroborate the situations or events that precede the Controller phenomenon, as identified in the theoretical phase. When asked about the antecedents of the concept, professionals frequently cited low adherence to safety protocols, along with a shortage of professionals specialized in HAI control. For the participants, these antecedents contribute to the underreporting of HAI cases in institutions.

The attributes identified in the theoretical phase align with the observed practices. The importance of updating the attribute “Implementation and management of the HAI Control Program” was highlighted, replacing the finding from the theoretical phase, considering that Brazilian Health Regulatory Agency (ANVISA in Portuguese) currently recommends HAI prevention and control based on this program. Another attribute widely discussed by professionals, and which corroborates the theoretical phase, is the continuous education of healthcare staff as the main focus for HAI control.

Although the findings from the literature regarding the consequences align with the field phase, the professionals, by consensus, saw the need to add the following consequences: cost reduction, adaptation of hospital infrastructure, and increased cleanliness of the environment. The inclusion of these three consequences was considered pertinent, as they are evident aspects of the practice of these professionals.

Thus, the pursuit of understanding the concept of Controller is justified by the need to carefully analyze the meaning of the term “Controller” for professionals directly involved in the control of these infections, based not only on literature data but also on the lived experiences of these professionals. Given the presented results and the conducted analysis, the following concept of HAI Controller was developed:

The HAI Controller is a duly trained healthcare professional responsible for disseminating measures to prevent and control HAI, promoting strategies to minimize infection rates in accordance with guidelines, to ensure the quality of healthcare and patient safety.

### Final Analytical Phase

The results of the analytical phase correspond to the interaction between the antecedents, attributes, and consequences from the theoretical and field phases. Thus, this phase involves the construction of the final product, which is the definition of the concept of HAI Controller. Chart 3 presents the essential elements of the concept definition found in both the theoretical and field phases. The elements added or adjusted after reaching consensus during the field phase are also included.

**Chart 3:** Essential elements of the concept definition found in the Theoretical and Field Phases, Natal, Rio Grande do Norte, Brazil, 2023

FINAL ANALYTICAL PHASE	
THEORETICAL PHASE	FIELD PHASE
**ANTECEDENTS:** • Few professionals specialized in HAI control • Low adherence of professionals to safety protocols • Increase in infection rates • Risk to patient safety	**ANTECEDENTS:** • Few professionals specialized in HAI control • Low adherence of professionals to safety protocols • Increase in infection rates • Risk to patient safety
**ATTRIBUTES:** • Realização da educação em saúde para prevenção e controle das IRAS • Implementação de diretrizes de prevenção e controle de IRAS • Identificação dos riscos de infecção, diagnóstico e notificação dos casos	**ATTRIBUTES:** • Provision of health education for the prevention and control of HAIs • Implementation and management of the HAI Control Program • Identification of infection risks, diagnosis, and case reporting
**CONSEQUENCES:** • Improvement in the quality of healthcare • Reduction and control of HAIs • Increased professional knowledge of their role	**CONSEQUENCES:** • Improvement in the quality of healthcare • Reduction and control of HAIs • Increased professional knowledge of their role • Cost reduction • Adaptation of hospital infrastructure • Increased cleanliness of the environment

It can be observed in Table 3 that modifications occurred between the previous phases. It is possible to identify that one attribute was adjusted during the field phase. It is believed that this discrepancy resulted from the updates brought by the professionals’ experience with the topic. It was found that three consequences, which were not identified during the theoretical phase, were added in the field phase. This change is possibly due to omissions in the analysis of manuscripts identified during the theoretical phase. Thus, all antecedents, attributes, and consequences were considered favorable in both phases. Therefore, all elements were taken into account for the development of the concept of the HAI Controller.

Having identified the essential elements and defined the concept of the HAI Controller, a model case was described based on the essential attributes of the concept to clarify it:

## MODEL CASE

M. F. G., 57 years old, Black, female, Brazilian, divorced, living alone in a community in the city of Natal (RN). According to the patient and her medical records, she was in her 9th postoperative day after a total hysterectomy at home when she developed fever, pain at the incision site, hyperemia, and purulent discharge from the surgical wound, which led her to return to the hospital. The surgery had been successful, and the patient remained in postoperative recovery in the hospital for 4 days before being discharged. Upon conducting the anamnesis and physical examination, the inpatient nurse identified signs and symptoms of infection, collected samples for culture, and notified the case to the CCIH. Through effective communication between the infection control committee and the care team regarding the possibility of colonization/infection, actions from the HAI Control Program were implemented and managed, such as the early adoption of contact isolation precautions until new cultures could identify the patient’s actual condition. In addition, health education was provided to the team on the proper use of personal protective equipment (PPE) and hand hygiene before delivering care. Staff rotation was minimized to prevent the spread of infection. The patient was also instructed on the importance of bed rest and not sharing personal items. As a result, with the appropriate actions carried out by the HAI controller nurse and the CCIH, effective and rapid progress was achieved not only in controlling the infection but also in treating the surgical wound, along with an investigation into the patient’s hospitalization to identify the source of the infection. The patient was discharged and continued her therapeutic treatment at home, gradually returning to her daily activities.

## DISCUSSION

### Theoretical Phase

Studies highlight the need for the regular updating of standards and protocols in healthcare institutions on this topic to ensure that the quality of healthcare remains excellent, in accordance with ANVISA’s recommendations^(10,11)^.

In Brazil, HAIs are widely discussed in studies as a terminology focused on the prevention and control of infections acquired in hospital settings and other healthcare institutions, as well as in legislation^(1)^.

Given the healthcare context in Brazil, HAI controllers recognize their role in pursuing quality care, even in the face of challenges, with the goal of advancing HAI control. This involves transition-ing from a clinical-epidemiological model, which focuses solely on retrospective data, to a more prospective and comprehensive model^(11)^.

In the hospital context, evidence shows cases of infections arising from ineffective practices in hospitals and even in outpatient services. Currently, due to the high risk of infection, hospitals and outpatient health services, such as medical clinics performing invasive procedures, are closely monitored^(12,13)^.

Regarding the identified antecedent, the literature emphasizes that the increasing complexity of healthcare services aimed at infection control requires well-trained professionals prepared to carry out the necessary actions. However, in practice, there is a significant challenge in finding qualified professionals for HAI control, which becomes a substantial obstacle to implementing the best practices recommended by ANVISA^(12)^.

The main attribute found in the research, corroborated by studies, highlights that health education is considered an effective strategy for professional qualification, in addition to encouraging professionals to be active agents in the infection control process, increasing their autonomy in decision-making. Due to low adherence to health education initiatives in services, this strategy has become a global problem of great magnitude^(14)^.

As for the primary consequence identified, authors point out that poor-quality care can lead to catastrophic outcomes. Therefore, care must be continuously monitored, ensuring that its purpose is the implementation of interventions aimed at patient safety^(15)^.

### Field Phase

Regarding the attribute “Implementation and management of the HAI Control Program,” the literature highlights that due to the significant health risks posed by hospital-acquired infections, Brazilian legislation has mandated that all hospitals in Brazil implement the HAI Control Program (PCIRAS)^(16)^. As for the attribute “Continuous Education,” the most effective way to prevent and control HAI is through the ongoing training of professionals and raising awareness of the importance of small actions in reducing the high rates of hospital infections. Health education initiatives help build knowledge among professionals, making them more committed and integrated into the work team^(17)^.

In relation to the consequences, the collaboration and mobilization of the entire multidisciplinary team are crucial to ensuring patient safety in the context of HAI. Any harm or risk to patient safety can increase healthcare costs, prolong hospital stays, and raise morbidity and mortality rates, leading to complications and, in extreme cases, death^(18)^.

### Study Limitations

This study faced limitations, including the absence of professionals from different fields due to scheduling conflicts, and the concept was not expanded to a global level, considering the sample of selected studies. Therefore, it is recommended that future studies be conducted to support the analysis of the concept from a broader perspective, taking other realities into account.

### Contributions to Nursing

The refinement of this new terminology’s definition may contribute to advancing knowledge on concept development techniques in nursing. Additionally, it plays an important role in technological development in health and nursing by bringing a new and improved concept into professional practice, which can be applied across the three levels of healthcare: primary, secondary, and tertiary. It is also believed that the evaluation, clarification, and refinement of the Controller concept will provide bedside nurses with a better understanding and awareness of their actions in controlling HAI.

## FINAL CONSIDERATIONS

This study enabled the definition of the concept of the HAI Controller, based on the dialogue between theoretical components and empirical evidence from the perspective of healthcare professionals working in the field. In the theoretical phase, four antecedents, three attributes, and three consequences were identified as essential elements for the concept. In the field phase, most professionals defined “Infection Controller” as the most appropriate term for this type of professional. The antecedents, attributes, and consequences identified in the theoretical phase were similar to those described by the professionals.

Based on the professionals’ understanding, there was a change in the attribute “Implementation and management of the HAI Control Program,” as well as the addition of three new antecedents. In the final analytical phase, it was observed that the elements identified in the previous phases were similar, although they had specific nuances, such as the antecedents allowing for the underreporting of HAI cases in institutions, and health education being highlighted as the main focus for controlling HAI. It is believed that by evaluating, clarifying, and refining the concept of the HAI Controller, this research will help nurses gain a better understanding of their role in providing care and increase their awareness of the necessary actions for controlling these infections.
