# Supplementary material for: Healthcare-Associated Infections Controller: Concept Development
Source: Rev Bras Enferm. 2025 Mar 10;78(1):e20230443. doi: 10.1590/0034-7167-2023-0443 (PMC11893053; doi:10.1590/0034-7167-2023-0443)
Supplement: Translation

# Translation: Controlador de Infecções Relacionadas à Assistência à Saúde: Formação de Conceito

**DOI:** 10.1590/0034-7167-2023-0443pt

**Authors:** Karolayne Cabral Matias, Cyntia Leenara Bezerra da Silva, Hanna Priscilla da Silva Medeiros, Maria Isabel da Conceição Dias Fernandes, Dândara Nayara Azevêdo Dantas, Lays Pinheiro de Medeiros, Allyne Fortes Vitor

**Affiliations:** I Universidade Federal do Rio Grande do Norte. Natal, Rio Grande do Norte, Brasil; II Hospital Universitário Ana Bezerra. Santa Cruz, Rio Grande do Norte, Brasil

**Keywords:** Formação de Conceito, Controle de Infecção, Infecção Hospitalar, Enfermagem, Atenção à Saúde

## Abstract

**Objetivo:**

Desenvolver o conceito de Controlador de Infecções Relacionadas à Assistência à Saúde.

**Método:**

Estudo metodológico qualitativo, fundamentado no Modelo Híbrido de Desenvolvimento de Conceito, realizado em três fases: Fase teórica (por meio de uma *scoping review*, com número amostral de 28 estudos), Fase de campo (encontro online com 30 profissionais da área) e Fase analítica final (análise dos dados e definição do conceito).

**Resultados:**

Os antecedentes, atributos e consequentes identificados na fase teórica foram semelhantes aos descritos pelos profissionais. Na fase de campo, a maioria dos profissionais definiu o “Controlador de infecção” como o termo mais adequado para esse profissional. Na fase analítica final, os elementos identificados nas fases anteriores são similares, entretanto, possuem particularidades.

**Considerações finais:**

Desenvolveu-se o conceito de Controlador de Infecções Relacionadas à Assistência à Saúde, subsidiando ao enfermeiro uma melhor compreensão sobre seu papel na assistência prestada e maior conscientização sobre suas ações.

## INTRODUÇÃO

Para atender efetivamente às necessidades do paciente, é essencial proporcionar qualidade na saúde, visando o bem-estar e o cuidado seguro. Prejuízos na qualidade do cuidado e na segurança do paciente predispõem a vários agravos, como as Infecções Relacionadas à Assistência à Saúde (IRAS). Anualmente, em todo o mundo, as IRAS afetam aproximadamente 1,5 milhão de pessoas, tornando-se um grande problema de saúde em nível mundial e uma das principais preocupações é a redução de sua incidência^(1,2)^.

Ao considerar a qualidade dos serviços relacionados ao controle e prevenção das IRAS, evidencia-se o impacto que os profissionais geram na segurança da assistência prestada, sendo necessário que se tenha conhecimento de suas ações^(3,4)^.

No contexto das IRAS, a Portaria 2.616/1998 do Ministério da Saúde indica a participação preferencial de um enfermeiro entre os membros executores dessas diretrizes, uma vez que essa profissão atua de forma ininterrupta na assistência direta ao indivíduo e realiza procedimentos invasivos e potencialmente contaminados. Consequentemente, o enfermeiro possui maior responsabilidade na profilaxia e no controle das infecções^(5,6)^.

Apesar de ser uma temática amplamente discutida na literatura em relação às suas origens e às ações gerais de prevenção e controle das IRAS, verifica-se baixo conhecimento e uma lacuna conceitual importante sobre o conceito de controlador de IRAS no Brasil. Isso pode estar contribuindo para a dificuldade de reconhecimento dos enfermeiros controladores em seu campo de atuação. Diante do exposto e da problemática apresentada, surgem as seguintes questões norteadoras: Quais são as definições conceituais de Controlador de IRAS? E, por fim, quais são os antecedentes, atributos e consequentes do conceito Controlador de IRAS?

## OBJETIVO

Desenvolver o conceito de Controlador de IRAS.

## MÉTODOS

### Aspectos éticos

A pesquisa obteve aprovação do Comitê de Ética em Pesquisa (CEP) da Universidade Federal do Rio Grande do Norte (UFRN). A participação dos controladores foi consolidada mediante aceitação e assinatura online do Termo de Consentimento Livre e Esclarecido ( TCLE), com informações asseguradas e esclarecidas sobre seus direitos. A privacidade e o sigilo das informações coletadas foram respeitados.

### Tipo de estudo

Trata-se de um estudo metodológico, de abordagem qualitativa, fundamentado no referencial teórico-metodológico do Modelo Híbrido de Desenvolvimento de Conceitos, proposto por Schwartz-Barcott e Kim^(7)^. Este modelo consiste em uma interação entre a análise teórica e a observação empírica da pesquisa de campo, permitindo ao pesquisador formular e refinar terminologias ainda em sua fase inicial de desenvolvimento, bem como detectar inconsistências e erros conceituais. O produto final é a definição do conceito. O modelo compreende três fases, que os autores sugerem que sejam sobrepostas em seu desenvolvimento: fase teórica, fase de campo e fase analítica final^(7)^.

### Procedimentos metodológicos

Como estratégia na fase teórica para coleta e análise de dados da literatura, foi realizada uma *Scoping Review*, utilizando o referencial metodológico do JBI^(8)^ e o modelo PRISMA para guiar todo o relato dos resultados, a fim de conferir maior rigor científico à revisão. A revisão foi operacionalizada por um protocolo de pesquisa. Realizou-se uma busca preliminar na literatura para verificar se já existiam estudos de revisão com o mesmo objetivo, não sendo identificados estudos anteriores.

Aplicou-se a estratégia PCC – População (profissionais da saúde), Conceito (Controlador) e Contexto (IRAS em nível mundial) e, ao correlacionar os tópicos-chave com os objetivos propostos, delimitaram-se as seguintes questões norteadoras da pesquisa: Qual é a origem, a descrição e os contextos do conceito de Controlador? Quais são as palavras ou expressões utilizadas na literatura para descrever o conceito de Controlador de IRAS? Quais são os antecedentes, atributos e consequentes do conceito de Controlador de IRAS?

Foram delimitados os descritores “Infecção Hospitalar; *Cross Infection*”, “Controle de Infecção; *Infection Control*”, “Enfermagem; *Nursing*” e “Atenção à Saúde; *Delivery of Health Care*” após consulta aos Descritores em Ciências da Saúde (DeCS) e *Medical Subject Headings* (MeSH). Os dados foram coletados nas seguintes fontes de dados eletrônicos: *Latin American and Caribbean Health Science Literature Database* (LILACS), SCOPUS (Elsevier), PubMed e *Cumulative Index to Nursing,* acessadas via Comunidade Acadêmica Federada (CAFe), por meio do portal de periódicos da Comissão de Aperfeiçoamento de Pessoal de Nível Superior (CAPES). Destaca-se que foi empregada a estratégia de busca avançada para todos os cruzamentos.

Para a seleção dos estudos, estabeleceram-se os seguintes critérios de inclusão: acessíveis na íntegra, nas fontes de dados elencadas, que abordassem o conceito de Controlador de IRAS, nos idiomas inglês, português e espanhol. Foram excluídos do estudo artigos de opinião, cartas ao editor e editoriais.

Através dos cruzamentos nas fontes de dados selecionadas, foram identificados 1.331 estudos. Destes, 291 foram excluídos por apresentarem duplicidade. Dessa forma, 982 artigos foram lidos pelos títulos e resumos, dos quais 924 foram retirados por não atenderem aos critérios de elegibilidade. Assim, 58 estudos foram lidos integralmente. Mediante busca reversa, não foram acrescentados artigos. Ao final, 28 estudos compuseram a amostra final da revisão.

Após serem tabulados em um banco de dados no programa Excel®, os resultados foram exportados para o programa estatístico *Statistical Package for the Social Sciences* 22.0 (SPSS) e analisados por meio de estatística descritiva simples. Todos os resultados obtidos foram apresentados em tabelas e quadros.

### Cenário do estudo

Foi realizada a análise de currículos existentes na Plataforma Lattes, do portal do Conselho Nacional de Desenvolvimento Científico e Tecnológico (CNPQ), e utilizou-se a técnica snowball sampling ou bola de neve para a seleção dos participantes. O recrutamento dos participantes se deu por convite via aplicativo de mensagens (WhatsApp), com o objetivo de participarem de um encontro para avaliarem as informações encontradas na fase teórica. O participante era orientado a clicar em um link e, ao fazê-lo, era redirecionado para uma página do *Google® Forms* contendo o TCLE em formato eletrônico.

### Fonte de dados

Foram convidados profissionais da área de controle de IRAS que atuam como membros executores, fazendo parte do serviço de controle de IRAS em seus três níveis, acreditando-se que o sucesso da técnica de grupo de consenso se dá pela qualificação dos participantes^(9)^. Foram excluídos do estudo os profissionais de saúde que não fazem parte do Serviço de Controle de Infecções Relacionadas à Assistência à Saúde (SCIRAS) ou que atuam na Comissão de Controle de Infecções Relacionadas à Assistência à Saúde (CCIRAS) apenas como membros consultores. No total, 30 profissionais participaram do estudo: 26 enfermeiros, 3 técnicos de enfermagem e 1 auxiliar administrativo da Comissão de Controle de Infecção Hospitalar (CCIH). Durante o processo de recrutamento, houve 80 recusas para participar da pesquisa.

### Coleta e organização dos dados

A coleta de dados ocorreu no mês de fevereiro de 2023, de forma remota, por meio da plataforma *Google Meets*, possibilitando que os controladores de IRAS, residentes em várias partes do país, participassem do encontro. Foi enviado aos participantes um link que redirecionava para a página do *Google Forms* contendo o termo de autorização para gravação de voz e imagem.

O instrumento de coleta de dados consistiu em uma apresentação estruturada, com os seguintes pontos: 1. Análise das expressões encontradas na literatura para definir esse profissional; 2. Análise do conceito desenvolvido; 3. Análise dos antecedentes do conceito; 4. Análise dos atributos do conceito; 5. Análise dos consequentes do conceito.

Os encontros foram guiados pela pesquisadora, que explicou cada etapa para que os controladores chegassem a um consenso sobre cada item teórico encontrado na etapa anterior, permitindo a observação atenta da pesquisadora. A análise dos dados ocorreu de forma descritiva, sendo transcrita e apreciada pela pesquisadora. Os itens que obtiveram consenso de 100% entre os controladores foram considerados validados. Os resultados obtidos foram apresentados em tabelas e quadros. A análise qualitativa dos dados foi realizada por meio da técnica de análise de conteúdo. Os participantes foram identificados por códigos de C1 a C30, garantindo que nenhuma informação pudesse revelar suas identidades.

### Fase Analítica Final

Embora o processo tenha sido apresentado de forma linear, a fase analítica permeou todo o desenvolvimento da pesquisa. As fases anteriores foram regularmente avaliadas de forma minuciosa para garantir a confiabilidade e o rigor metodológico que o modelo requer. Dessa forma, com a integração das fases, foi possível construir uma estrutura conceitual para o fenômeno Controlador, exposta em forma de texto.

A revisão focou em ajustes gramaticais, de concordância e ortográficos para melhorar a clareza do texto sem alterar o conteúdo ou a formatação das citações.

## RESULTADOS

### Fase Teórica

Nesta fase, foram investigadas a origem, definições, antecedentes, atributos e consequentes do conceito de Controlador de IRAS, por meio de uma scoping review, resultando em uma amostra final de 28 estudos.

Nos estudos analisados, observou-se um padrão crescente de publicações nos últimos 10 anos, com prevalência nos anos de 2018 a 2022 (39,3%). Identificou-se também que a maioria dos estudos foi desenvolvida no Brasil (96,4%) e que 89,3% foram publicados em português.

Quanto à origem do conceito de Controlador de IRAS, foi possível identificar não apenas a natureza da utilização do conceito, mas também os elementos importantes que permitiram sua evolução ao longo do tempo. Conforme evidenciado, a busca pela qualidade nos serviços de saúde associados às IRAS é o principal acontecimento pioneiro no surgimento do conceito (25%).

As definições do conceito investigadas nesta fase serviram como base para a construção do conceito, em conjunto com a perspectiva multiprofissional dos controladores de IRAS, desenvolvida na fase de campo. O Quadro 1 apresenta a síntese do conceito de Controlador de IRAS já publicado na literatura.

**Quadro 1:** Síntese das definições do conceito Controlador de IRAS encontradas nos estudos da *Scoping review*, Natal, Rio Grande do Norte, Brasil, 2023

Definições de Controlador de IRAS	Número de estudos	Identificação das referências
Responsável por supervisionar normas e rotinas, capacitar funcionários e profissionais, racionalizar o uso de antimicrobianos, fornecer informações epidemiológicas e minimizar o índice das IRAS.	11	1, 4, 5,7, 9, 11, 14, 15, 16, 19, 24
Implementador de medidas de prevenção e controle sistemático da infecção e redutor dos riscos e dos danos, de modo que seu gerenciamento seja seguro.	8	2, 3, 20, 21, 22, 23
Responsável por identificar e diagnosticar os diferentes tipos de infecções hospitalares, junto das ações de manutenção de boas práticas de prevenção, controle e informação sobre as infecções.	5	6, 8, 13, 17, 27
Desenvolvedor de medidas, que normatizam a organização das ações e os serviços de saúde, em consonância com os princípios e diretrizes da prevenção e controle das infecções, assim possibilita a minimização do seu potencial iatrogênico.	3	10, 18, 28
Gerenciador do processo de trabalho, ao planejar e executar ações de controle de IRAS e auxílio nas ações de melhorias na área.	1	12

Observou-se que os estudos selecionados pela revisão utilizavam sete expressões distintas para descrever o conceito de Controlador, a saber: “Supervisor” (21,4%); “Responsável por prevenir” (17,9%); “Gerenciador de danos e riscos” e “Minimizador de potencial de infecção” (14,3% cada); “Controlador de Infecção”, “Estrategista na implementação de boas práticas” e “Identificador de suscetibilidades e vulnerabilidades” (10,7% cada). Destaca-se que todas as expressões identificadas apresentavam similaridade em suas ações.

Com o desenvolvimento da análise da *scoping review*, viu-se a necessidade de determinar os aspectos contextuais (prático e populacional) que contribuíram para a interpretação e caracterização do conceito. Observou-se que o conceito é utilizado mais frequentemente no contexto hospitalar (53,6%).

Os elementos essenciais do conceito foram derivados em quatro antecedentes, três atributos e três consequentes. A Tabela 1 demonstra a categorização desses elementos essenciais.

**Tabela 1:** Elementos essenciais do conceito Controlador apontados na literatura pesquisada. Natal, Rio Grande do Norte, Brasil, 2023

Elementos essenciais do conceito Controlador	n	%
Antecedentes		
Poucos profissionais especializados para o controle de IRAS	14	50
Pouca adesão dos profissionais aos protocolos de segurança	8	28,6
Aumento das taxas de Infecção	4	14,3
Risco à Segurança do Paciente	2	7,1
Atributos		
Realização da educação em saúde para prevenção e controle das IRAS	14	50
Implementação de diretrizes de prevenção e controle de IRAS	8	28,6
Identificação dos riscos de infecção, diagnóstico e notificação dos casos	6	21,4
Consequentes		
Melhora da qualidade da assistência à saúde	13	46,4
Redução e controle das IRAS	9	32,1
Aumento do conhecimento do profissional sobre seu papel	6	21,4

Como antecedente mais frequente do conceito de Controlador, destaca-se a escassez de profissionais especializados para o controle de IRAS (50%). O principal atributo encontrado na literatura foi a “realização da educação em saúde para prevenção e controle das IRAS” (50%). A melhora da qualidade da assistência à saúde foi identificada como a principal consequência (46,4%).

Com o delineamento teórico mais claro, foi possível proceder à fase de campo de maneira mais precisa. Além disso, foi elaborada a seguinte definição de Controlador de IRAS, para orientar os profissionais na busca pelo refinamento do conceito: “Profissional da saúde devidamente capacitado, responsável pela execução de medidas para prevenção e controle de IRAS, promovendo estratégias para a minimização do índice de infecções em consonância com as diretrizes, a fim de garantir a qualidade da assistência à saúde e a segurança do paciente.”

### Fase de Campo

A pesquisa de campo foi desenvolvida com a participação de 26 enfermeiros (86,7%), três técnicos de enfermagem (10%) e um auxiliar administrativo da CCIH (3,3%). Dos profissionais que participaram do estudo, 28 (93,3%) eram do sexo feminino, com idade média de 35,9 anos. Em relação à formação acadêmica, 21 (70%) possuíam algum tipo de especialização e o tempo médio de formação foi de 10,3 anos. No que se refere à atuação profissional, sete (23,3%) declararam exercer suas funções em Natal, no Rio Grande do Norte, com uma média de 5,5 anos de tempo de atuação. A Tabela 2 detalha as características dos participantes do estudo.

**Tabela 2:** Caracterização dos profissionais que participaram do estudo. Natal, Rio Grande do Norte, Brasil, 2023

Variáveis	n		%
Profissão			
Enfermeiro	26		86,7
Técnico de enfermagem	3		10
Auxiliar administrativo	1		3,3
Total	30		100%
Sexo			
Feminino	28		93,3
Masculino	2		6,7
Total	30		100%
Formação acadêmica			
Especialização	11		36,7
Graduação	9		30
Mestrado	7		23,3
Curso técnico	3		10
Total	30		100%
**Variáveis**	**Média**	**Desvio padrão**	**Mediana**
Idade*	35,9	9,3	33,5
Tempo de formação*	10,3	8,0	8,5
Tempo de atuação*	5,5	4,7	4,5

Legenda: *Em anos.

No contexto prático, todos os profissionais afirmaram que atuam ou já atuaram em hospitais (100%). No contexto populacional, destaca-se o Serviço de Controle de Infecção Hospitalar, com 12 profissionais (40%), seguido da Comissão de Controle de Infecção Hospitalar, com 10 profissionais (33,3%). Nenhum dos profissionais relatou atuar ou ter atuado em ambientes virtuais. No que diz respeito à mensuração do grau de familiaridade dos participantes com o objeto de estudo, 29 profissionais (96,7%) relataram já ter ouvido a expressão “Controlador” anteriormente.

Os elementos do conceito encontrados na literatura para definir o Controlador de IRAS foram apresentados aos profissionais nesta fase de coleta de dados. Em relação às expressões delimitadas, 20 deles (66,7%) acreditam que “Controlador de infecção” é também um termo adequado para definir esse tipo de profissional; 11 (36,7%) selecionaram as opções “Supervisor” e “Estrategista na implementação de boas práticas”, 10 (33,4%) escolheram “Gerenciador de danos e riscos”, e 4 (13,4%) escolheram “Responsável por prevenir”. Assim, em consenso, os participantes escolheram a expressão “Controlador de infecção” como a mais adequada para o conceito.

Quanto aos antecedentes, atributos e consequentes observados nesta fase, estão descritos no Quadro 2. Os elementos identificados nesta fase, semelhantes àqueles identificados na fase teórica, estão expostos sem realce. Novos elementos ou mudanças que emergiram da compreensão e significados expressados pelos profissionais foram destacados em negrito.

**Quadro 2:** Elementos essenciais do conceito Controlador identificados na pesquisa de campo. Natal, Rio Grande do Norte, Brasil, 2023

**ANTECEDENTES**	Poucos profissionais especializados para o controle de IRAS
	Pouca adesão dos profissionais aos protocolos de segurança
	Aumento das taxas de infecção
	Risco a segurança do paciente
**ATRIBUTOS**	Realização da educação em saúde para prevenção e controle das IRAS
	Implementação e gerenciamento do Programa de controle de IRAS
	Identificação dos riscos de infecção, diagnóstico e notificação dos casos
**CONSEQUENTES**	Melhora da qualidade da assistência à saúde
	Redução e controle das IRAS
	Aumento do conhecimento do profissional sobre seu papel
	Redução de custos
	Adequação da estrutura hospitalar
	Aumento da limpeza do ambiente

Na avaliação final do conceito, observou-se que os elementos identificados nas fases anteriores são similares, contudo, apresentam particularidades que foram discutidas ao longo do estudo. Através da fase de campo, foi possível corroborar as situações ou eventos que antecedem o fenômeno Controlador, identificados na fase teórica. Quando questionados sobre os antecedentes do conceito, os profissionais citaram com frequência a baixa adesão aos protocolos de segurança, juntamente com a escassez de profissionais especializados para o controle de IRAS. Para os participantes, esses antecedentes favorecem a subnotificação dos casos de IRAS nas instituições.

Os atributos identificados na fase teórica estão em consonância com as práticas observadas. Foi destacada a importância da atualização do atributo “Implementação e gerenciamento do Programa de Controle de IRAS”, substituindo o achado na fase teórica, considerando que, atualmente, a Agência Nacional de Vigilância Sanitária – Anvisa, preconiza a prevenção e controle de IRAS com base nesse programa. Outro atributo bastante discutido pelos profissionais, que corrobora a fase teórica, é a realização da educação continuada como foco principal para o controle das IRAS.

Embora os achados da literatura sobre os consequentes estejam em consonância com a fase de campo, os profissionais, em consenso, viram a necessidade de acrescentar os seguintes consequentes: Redução de custos, Adequação da estrutura hospitalar e Aumento da limpeza do ambiente. A inclusão desses três antecedentes foi considerada pertinente, pois são aspectos evidentes na prática desses profissionais.

Dessa forma, justifica-se a busca pela compreensão do conceito de Controlador pela necessidade de analisar criteriosamente o significado da terminologia “Controlador” para os profissionais que atuam diretamente no controle dessas infecções, baseando-se não apenas em dados da literatura, mas também nas experiências vividas por esses profissionais. Diante dos resultados apresentados e da análise realizada, produziu-se o seguinte conceito de Controlador de IRAS:

O Controlador de IRAS é o profissional da saúde devidamente capacitado, responsável por disseminar medidas para prevenção e controle de IRAS, promovendo estratégias para a minimização do índice de infecções, em consonância com as diretrizes, a fim de garantir a qualidade da assistência à saúde e a segurança do paciente.

### Fase Analítica Final

Os resultados da fase analítica correspondem à interação entre os antecedentes, atributos e consequentes das fases teórica e de campo. Assim, essa fase trata-se da construção do produto final da definição do conceito Controlador de IRAS. O Quadro 3, apresenta os elementos essenciais de definição do conceito encontrados na fase teórica e na fase de campo. Foram reajustados e acrescentados os elementos inseridos após consenso em fase de campo.

**Quadro 3:** Elementos essenciais de definição do conceito em estudo encontrados na Fase Teórica e na Fase de Campo, Natal, Rio Grande do Norte, Brasil, 2023

FASE ANALÍTICA FINAL	
FASE TEÓRICA	FASE DE CAMPO
**ANTECEDENTES:** • Poucos profissionais especializados para o controle de IRAS • Pouca adesão dos profissionais aos protocolos de segurança • Aumento das taxas de infecção • Risco a segurança do paciente	**ANTECEDENTES:** • Poucos profissionais especializados para o controle de IRAS • Pouca adesão dos profissionais aos protocolos de segurança • Aumento das taxas de infecção • Risco a segurança do paciente
**ATRIBUTOS:** • Realização da educação em saúde para prevenção e controle das IRAS • Implementação de diretrizes de prevenção e controle de IRAS • Identificação dos riscos de infecção, diagnóstico e notificação dos casos	**ATRIBUTOS:** • Realização da educação em saúde para prevenção e controle das IRAS • Implementação e gerenciamento do Programa de controle de IRAS • Identificação dos riscos de infecção, diagnóstico e notificação dos casos
**CONSEQUENTES:** • Melhora da qualidade da assistência à saúde • Redução e controle das IRAS • Aumento do conhecimento do profissional sobre seu papel	**CONSEQUENTES:** • Melhora da qualidade da assistência à saúde • Redução e controle das IRAS • Aumento do conhecimento do profissional sobre seu papel • Redução de custos • Adequação da estrutura hospitalar • Aumento da limpeza do ambiente

Observa-se no Quadro 3 que ocorreram modificações entre as fases anteriores. É possível identificar que um atributo foi reajustado na fase de campo. Acredita-se que essa discordância seja decorrente da atualização trazida pela experiência dos profissionais para a temática. Verificou-se que três consequentes, que não foram encontrados na fase teórica, foram inseridos na fase de campo. Essa alteração se deve, possivelmente, a omissões nas análises dos manuscritos identificados na fase teórica. Dessa forma, todos os antecedentes, atributos e consequentes foram considerados favoráveis nas duas fases. Assim, todos foram levados em conta para o desenvolvimento do conceito de Controlador de IRAS.

Tendo sido identificados os elementos essenciais e a definição do conceito de Controlador de IRAS, descreveu-se um caso modelo, baseado nos atributos essenciais do conceito, a fim de clarificá-lo:

## CASO MODELO

M. F. G., 57 anos, negra, sexo feminino, brasileira, divorciada, mora sozinha em uma comunidade do município de Natal (RN). Segundo informações da paciente e também de seu prontuário médico, ela estava em seu 9º dia de pós-operatório de histerectomia total, em casa, quando apresentou febre, dor no local da incisão, hiperemia e secreção purulenta na ferida operatória, decidindo retornar ao hospital. A cirurgia havia sido bem-sucedida, e a paciente permaneceu em repouso pós-operatório no hospital por 4 dias, recebendo alta em seguida. Ao realizar anamnese e exame físico, a enfermeira de internação identificou sinais e sintomas de infecção, colheu amostras para cultura e notificou o caso à CCIH. Pela comunicação eficaz entre a comissão de controle de infecção e a equipe assistencial sobre a possibilidade de colonização/ infecção, foram implementadas e gerenciadas ações do Programa de Controle de IRAS, como a adoção precoce de precauções de isolamento por contato até que novas culturas identificassem o real estado da paciente. Além disso, realizou-se educação em saúde com a equipe sobre o uso adequado de EPIs, a higienização das mãos antes da prestação de cuidados, e foi feito o dimensionamento para diminuição da rotatividade de profissionais, a fim de evitar a disseminação da infecção. A paciente também foi orientada sobre a importância da restrição ao leito e o não compartilhamento de objetos pessoais. Dessa forma, com as ações adequadas realizadas pela enfermeira controladora de IRAS, junto à CCIH, foi possível um progresso eficaz e ágil não só no controle da infecção, mas também no tratamento da ferida operatória, além da investigação da internação da paciente para identificar a origem da infecção. Assim, a paciente recebeu alta e continuou seu tratamento terapêutico em casa, retornando gradativamente às suas atividades diárias.

## DISCUSSÃO

### Fase Teórica

Estudos apontam a necessidade de que a atualização de normas e protocolos nas instituições de saúde acerca da temática em questão se torne uma prática corriqueira, para que a qualidade da assistência à saúde continue sendo de excelência, seguindo as recomendações da ANVISA^(10,11)^.

No Brasil, as IRAS são amplamente discutidas nos estudos como uma terminologia voltada para a prevenção e controle de infecções adquiridas em ambiente hospitalar e demais instituições de saúde, bem como na legislação^(1)^.

Diante do cenário da assistência no Brasil, os profissionais controladores de IRAS reconhecem seu papel na busca pela qualidade da assistência, mesmo frente às dificuldades enfrentadas, com o objetivo de avançar na perspectiva do controle de IRAS. Isso implica uma transição de um modelo clínico-epidemiológico, que foca apenas em dados retrospectivos, para um modelo mais prospectivo e abrangente^(11)^.

No contexto hospitalar, evidências mostram casos de infecções decorrentes de práticas ineficazes em hospitais e até mesmo em serviços ambulatoriais. Atualmente, devido ao alto risco de infecção, hospitais e serviços ambulatoriais de saúde, como clínicas médicas que realizam procedimentos invasivos, são criteriosamente observados^(12,13)^.

Quanto ao antecedente identificado, os dados da literatura enfatizam que a crescente complexidade dos serviços de saúde voltados para o controle de infecções demanda profissionais devidamente capacitados e prontos para desenvolver as ações necessárias. No entanto, o que se observa na prática é uma grande dificuldade em encontrar profissionais qualificados para o controle de IRAS, o que se torna um obstáculo significativo para a implementação das boas práticas recomendadas pela ANVISA^(12)^.

O principal atributo encontrado na pesquisa, corroborado por estudos, evidencia que a educação em saúde é considerada uma estratégia eficaz na qualificação do profissional, além de incentivar o profissional a ser um agente atuante no processo de controle de infecções, aumentando sua autonomia nas decisões de forma assertiva. Devido à baixa adesão às ações de educação em saúde nos serviços, essa estratégia acaba por se tornar um problema de grande magnitude em nível mundial^(14)^.

Quanto à principal consequência identificada, autores apontam que a má qualidade da assistência pode resultar em consequências catastróficas. Assim, a assistência deve ser constantemente monitorada, de modo que sua finalidade seja a implementação de intervenções voltadas para a segurança do paciente^(15)^.

### Fase de campo

No que se refere ao atributo “Implementação e gerenciamento do Programa de Controle de IRAS”, a literatura destaca que, devido às infecções hospitalares constituírem riscos significativos à saúde, a legislação brasileira estabeleceu que todos os hospitais do Brasil devem implantar o Programa de Controle de Infecções Relacionadas à Assistência à Saúde (PCIRAS)^(16)^.

Quanto ao atributo “Educação Continuada”, a forma mais eficaz de prevenir e controlar as IRAS é por meio da capacitação contínua dos profissionais e da conscientização sobre a importância das pequenas ações para a redução dos altos índices de infecções hospitalares. A realização de ações voltadas para a educação em saúde favorece a construção do conhecimento entre os profissionais, tornando-os comprometidos e integrados à equipe de trabalho^(17)^.

Em relação aos consequentes, é crucial a colaboração e mobilização de toda a equipe multidisciplinar para garantir a segurança do paciente no que tange às IRAS. Qualquer dano ou risco à segurança do paciente pode aumentar os custos com a saúde, prolongar o tempo de internação e elevar os índices de morbimortalidade, ocasionando complicações e, em casos extremos, o óbito^(18)^.

### Limitações do estudo

O presente estudo apresentou como limitações a ausência de profissionais de diferentes áreas, devido à indisponibilidade de horário, e a não ampliação do uso desse conceito para o nível mundial, considerando a amostra dos estudos selecionados. Recomenda-se, portanto, que estudos futuros sejam desenvolvidos para apoiar a análise do conceito sob uma perspectiva mais ampla, levando em consideração outras realidades.

### Contribuições para a Enfermagem

A adequação da definição dessa nova terminologia pode contribuir para o avanço do conhecimento sobre técnicas de desenvolvimento de conceitos em Enfermagem. Além disso, desempenha um importante papel no desenvolvimento tecnológico em saúde e enfermagem, ao trazer para a prática profissional um conceito novo e aperfeiçoado, que pode ser aplicado nos três níveis de atenção à saúde: primário, secundário e terciário. Acredita-se também que a avaliação, clarificação e refinamento do conceito de Controlador poderão proporcionar ao enfermeiro assistencial uma melhor compreensão e conscientização sobre suas ações no controle das IRAS.

## CONSIDERAÇÕES FINAIS

O presente estudo permitiu a definição do conceito de Controlador de IRAS, com base na interlocução entre componentes teóricos e evidências empíricas, sob a ótica dos profissionais de saúde que atuam na área. Na fase teórica, foram detectados quatro antecedentes, três atributos e três consequentes, todos elementos essenciais para o conceito. Já na fase de campo, a maioria dos profissionais definiu o “Controlador de infecção” como o termo mais adequado para esse tipo de profissional. Os antecedentes, atributos e consequentes identificados na fase teórica foram semelhantes aos descritos pelos profissionais.

Com base na compreensão dos profissionais, houve uma mudança no atributo “Implementação e gerenciamento do Programa de Controle de IRAS”, bem como a adição de três novos antecedentes. Na fase analítica final, observou-se que os elementos identificados nas fases anteriores são similares, embora possuam particularidades, como o fato de os antecedentes abrirem espaço para a subnotificação de casos de IRAS nas instituições e a educação em saúde ser destacada como foco principal para o controle das IRAS. Acredita-se que esta pesquisa, ao avaliar, clarificar e refinar o conceito de Controlador de IRAS, irá subsidiar os enfermeiros para uma melhor compreensão de seu papel na assistência prestada e aumentar sua conscientização sobre as ações necessárias para o controle dessas infecções.
